# Distribution of hybrid entanglement and hyperentanglement with time-bin for secure quantum channel under noise via weak cross-Kerr nonlinearity

**DOI:** 10.1038/s41598-017-09510-9

**Published:** 2017-08-31

**Authors:** Jino Heo, Min-Sung Kang, Chang-Ho Hong, Hyung-Jin Yang, Seong-Gon Choi, Jong-Phil Hong

**Affiliations:** 1 College of Electrical and Computer Engineering, Chungbuk National University, Chungdae-ro 1, Seowon-Gu, Cheongju Republic of Korea; 2 Center for Quantum Information, Korea Institute of Science and Technology (KIST), Seoul, 136-791 Republic of Korea; 3 National Security Research Institute, P.O. Box 1, Yuseong, Daejeon 34188 Republic of Korea; 4 Department of Physics, Korea University, Sejong, 339-700 Republic of Korea

## Abstract

We design schemes to generate and distribute hybrid entanglement and hyperentanglement correlated with degrees of freedom (polarization and time-bin) via weak cross-Kerr nonlinearities (XKNLs) and linear optical devices (including time-bin encoders). In our scheme, the multi-photon gates (which consist of XKNLs, quantum bus [qubus] beams, and photon-number-resolving [PNR] measurement) with time-bin encoders can generate hyperentanglement or hybrid entanglement. And we can also purify the entangled state (polarization) of two photons using only linear optical devices and time-bin encoders under a noisy (bit-flip) channel. Subsequently, through local operations (using a multi-photon gate via XKNLs) and classical communications, it is possible to generate a four-qubit hybrid entangled state (polarization and time-bin). Finally, we discuss how the multi-photon gate using XKNLs, qubus beams, and PNR measurement can be reliably performed under the decoherence effect.

## Introduction

Entanglement is significantly involved in quantum information processing schemes, such as quantum communications^1–3^, quantum computations^4–6^, and quantum networks^7–9^. Feasible and efficient quantum information processing schemes depend on the experimental suitability of realization and the efficient conservation of the correlations in entangled states. Thus, the generation and distribution of various types of entanglement should be investigated to acquire both experimental implementation and efficiency.

Hybrid entanglement is correlated between one type (i.e. polarization, spin, etc.) of degree of freedom (DOF) in company with another type of DOF (i.e. spatial mode, time-bin, etc.), such as entanglement with spatial mode, polarization, linear momentum, and spin^10, 11^; polarization/linear momentum of a single photon^12, 13^; polarization/angular momentum of a single photon^14^; and path-spin of a single neutron^15^ in theory and in practice^12–17^. Since it is possible to be correlated within a single particle, the quantum information processing schemes (such as entanglement swapping^18^, quantum key distribution^19^, and quantum teleportation)^20–22^, using hybrid entanglement as resources, can consume fewer resources than the technique utilizing only a single type of DOF.

Besides, for improvement of the channel capacity^23, 24^, hyperentanglement refers to the entanglement of a single system having correlations with several DOFs^25^. Research has been proposed into hyperentanglement due to diverse types of DOFs, such as polarization and momentum^26^, polarization and orbital angular momentum^14^, and time-bin^27, 28^. Many schemes based on the merits of hyperentanglement have been researched, as follows: superdense coding^14^, purification of (and eliminating noise of) entanglement^29–34^, analysis of the Bell state^26, 27, 35^, and quantum communications^36, 37^.

For a reliable quantum-controlled gate to enhance the performance of quantum information processing, indirect interaction between photons based on quantum non-demolition measurement can be assisted by optical nonlinearities. Cross-Kerr nonlinearities (XKNLs) in particular have been widely utilized for feasible optical multi-qubit gates^21, 22, 38–52^. But the decoherence effect of optical multi-qubit gates is inevitable in optical fiber due to loss of photons in practice. Some research^40–42, 46, 52, 53^ demonstrates that if homodyne measurements are used in optical multi-qubit gates, the fidelity of optical gates will decrease by evolving the output states into mixed states. Fortunately, by applying photon-number-resolving (PNR) measurements^54–57^, and a displacement operator^41, 42^ or quantum bus (qubus) beams^52^ with the increasing amplitude of the coherent state, the decoherence effect can be made arbitrarily small^41, 42, 52^.

In this paper, we propose schemes to generate and distribute hybrid entanglement and hyperentanglement between DOFs for polarization and time-bin using optical multi-qubit gates, which utilize XKNLs, qubus beams, and PNR measurement to obtain efficiency and robustness under the decoherence effect, and linear optical devices (including time-bin encoders). First, we present how a hybrid entangled state is correlated with two DOFs (polarization and time-bin) of two photons from Trent having a single multi-photon gate (via XKNLs), and with the process of verifying the quantum channel between Trent and users (Alice and Bob). Then, the users can reconstruct hybrid entanglement (polarization and time-bin) using time-bin encoders after verifying the participants. Also, we show the distribution and generation of hyperentanglement, having its own correlations of two DOFs (polarization and time-bin) of two photons, using two multi-photon gates (via XKNLs) and linear optical devices. Second, under a noisy channel (bit-flip noise), we introduce the purification of entanglement (correlated with only polarizations of two photons), as described by Li *et al*.^31^, from a noisy quantum state via only time-bin encoders and linear optical devices without nonlinear optical processes (XKNLs). And the local applications of nonlinear optical gates and time-bin encoders can extend to a four-photon hybrid entangled state from the purified two-photon entangled state through classical communications. Finally, we discuss the efficiency and performance of the multi-photon gate, based on XKNLs, qubus beams, and PNR measurement, under the decoherence effect^41, 42, 52^.

## Two-photon gates using XKNLs, and time-bin encoders via linear optical devices

**Figure 1:**
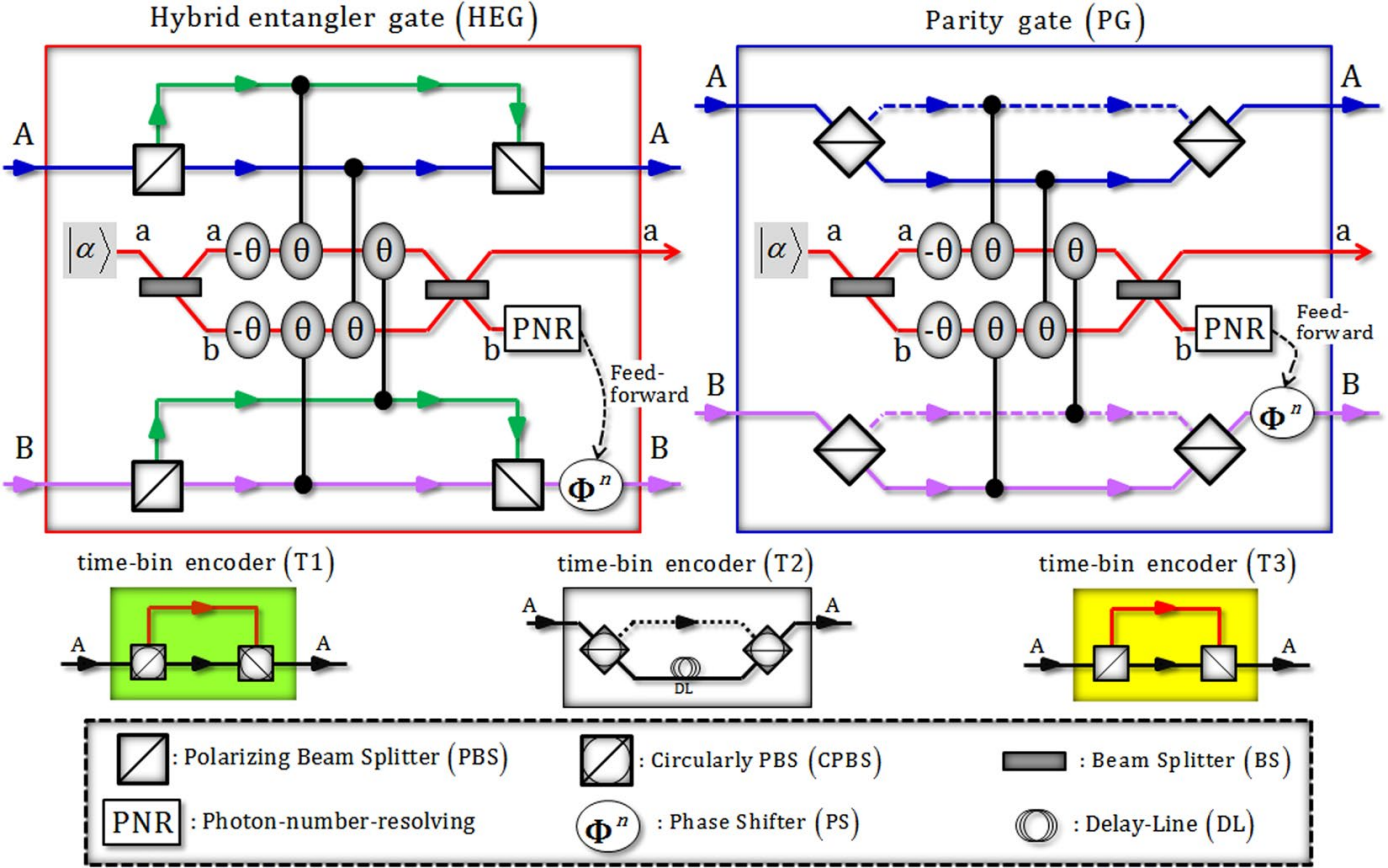
Plot schematically representing two-photon gates, a hybrid entangler gate (HEG) and a parity gate (PG), using XKNL, qubus beams, and PNR measurement and time-bin encoders (T1, T2, and T3) using linear optical devices. The HEG and the PG can generate a hybrid entangled state with two DOFs (polarization and time-bin) and an entangled state with one DOF (polarization) of two photons, respectively. The polarization of a single photon can be encoded with a relative time delay in terms of polarization by the difference in the lengths of the optical paths or delay-line (DL) in T1, T2, and T3.

We introduce the XKNL effect. The XKNL’s Hamiltonian is given as ${H}_{Kerr}=\hslash \chi {N}_{1}{N}_{2}$, where *N*_*i*_ and *χ* are the photon number operator and the strength of nonlinearity in the Kerr medium. If we consider ${|n\rangle }_{i}$ (signal state: *n* means the number of photons) and ${|\alpha \rangle }_{j}$ (coherent state: probe beam), the signal-probe system’s state is transformed to ${U}_{Kerr}{|n\rangle }_{1}{|\alpha \rangle }_{2}={e}^{i\theta {N}_{1}{N}_{2}}{|n\rangle }_{1}{|\alpha \rangle }_{2}={|n\rangle }_{1}{|\alpha {e}^{in\theta }\rangle }_{2}$ after the interactions in the Kerr medium, where $\theta =\chi t$ and *t* is the interaction time (conditional phase shift). And the linear phase shifter, which can rotate the coherent state in phase space by phase shifting operator (*U*_*PS*_), is expressed as ${U}_{PS}|\alpha \rangle ={e}^{i\theta N}|\alpha \rangle =|\alpha {e}^{i\theta }\rangle$. The optical gates shown in Fig. 1 are utilized in our schemes to generate and distribute hybrid entanglement and hyperentanglement between DOFs of polarization and time-bin under noisy channels (including the decoherence effect in the nonlinear optical gates). Here, we define the relations of the circularly polarized states and the linearly polarized states as $|R\rangle \equiv (|H\rangle +|V\rangle )/\sqrt{2}$ and $|L\rangle \equiv (|H\rangle -|V\rangle )/\sqrt{2}$.

### Hybrid entangler gate (HEG) using XKNLs, qubus beams, and PNR measurement

The HEG consists of four polarizing beam splitters (PBSs: transmit $|H\rangle$ and reflect $|V\rangle$), conditional phase shifts θ (four XKNLs: only positive phase) in Kerr media, two linear phase shifters (−θ), and two beam splitters (BSs) in qubus beams. For the generation of correlation (polarization) and simultaneous time-bin of the two photons (hybrid entanglement: two DOFs of polarization and time-bin), we can adjust the paths regarding two linear polarizations $|H\rangle$ and $|V\rangle$ of two photons by PBSs, such as $|V\rangle$ obtaining the time interval *l* in the long length of the optical path and $|H\rangle$ obtaining the time interval *s* over the short length of the optical path in the HEG, as described in Fig. 1. After the input state (${|R\rangle }_{1}^{{\rm{A}}}{|R\rangle }_{2}^{{\rm{B}}}\otimes {|\alpha \rangle }^{{\rm{a}}}$) passes through the PBSs, the Kerr media (XKNLs), and the BSs, the output state $|{{\boldsymbol{\psi }}}_{{\rm{HEG}}}\rangle$ is transformed as follows:

1 $$\begin{array}{cc}|{{\boldsymbol{\psi }}}_{{\rm{H}}{\rm{E}}{\rm{G}}}\rangle\, & =\,\frac{1}{\sqrt{2}}[{|\alpha \rangle}^{{\rm{a}}}\otimes \frac{1}{\sqrt{2}}({|H\rangle}_{1}^{{\rm{A}}}{|s\rangle}_{1}{|V\rangle}_{2}^{{\rm{B}}}{|l\rangle}_{2}\otimes {|0\rangle}^{{\rm{b}}}+{|V\rangle}_{1}^{{\rm{A}}}{|l\rangle}_{1}{|H\rangle}_{2}^{{\rm{B}}}{|s\rangle}_{2}\otimes {|0\rangle}^{{\rm{b}}})\\  & \quad +\,{|\alpha \cos \theta \rangle}^{{\rm{a}}}\otimes \frac{1}{\sqrt{2}}({|V\rangle}_{1}^{{\rm{A}}}{|l\rangle}_{1}{|V\rangle}_{2}^{{\rm{B}}}{|l\rangle}_{2}\otimes {|i\alpha \sin \theta \rangle}_{{\rm{b}}}+{|H\rangle}_{1}^{{\rm{A}}}{|s\rangle}_{1}{|H\rangle}_{2}^{{\rm{B}}}{|s\rangle}_{2}\otimes {|-i\alpha \sin \theta \rangle}^{{\rm{b}}})]\\  & =\,{|\alpha \rangle}^{{\rm{a}}}\otimes \frac{1}{\sqrt{2}}[\frac{1}{\sqrt{2}}({|H\rangle}_{1}^{{\rm{A}}}{|s\rangle}_{1}{|V\rangle}_{2}^{{\rm{B}}}{|l\rangle}_{2}+{|V\rangle}_{1}^{{\rm{A}}}{|l\rangle}_{1}{|H\rangle}_{2}^{{\rm{B}}}{|s\rangle}_{2})]\otimes {|0\rangle}^{{\rm{b}}}\\  & \quad +\,{|\alpha \cos \theta \rangle}^{{\rm{a}}}\otimes \frac{1}{\sqrt{2}}\cdot {e}^{-{(\alpha \sin \theta )}^{2}/2}\sum _{n=0}^{{\rm{\infty }}}\frac{{(i\alpha \sin \theta )}^{n}}{\sqrt{n!}}[\frac{1}{\sqrt{2}}({|V\rangle}_{1}^{{\rm{A}}}{|l\rangle}_{1}{|V\rangle}_{2}^{{\rm{B}}}{|l\rangle}_{2}+{(-1)}^{n}{|H\rangle}_{1}^{{\rm{A}}}{|s\rangle}_{1}{|H\rangle}_{2}^{{\rm{B}}}{|s\rangle}_{2})]\otimes {|n\rangle}^{{\rm{b}}},\end{array}$$

where $|{s}\rangle$ and $|{l}\rangle$ represent the early (passing short path) and late (passing long path) time bins. The BS transforms ${|\alpha \rangle }^{{\rm{a}}}{|\beta \rangle }^{{\rm{b}}}$ into ${|(\alpha +\beta )/\sqrt{2}\rangle }^{{\rm{a}}}{|(\alpha -\beta )/\sqrt{2}\rangle }^{{\rm{b}}}$, and $|\pm i\alpha \,\sin \,\theta \rangle ={e}^{-{(\alpha \sin \theta )}^{2}/2}\sum _{n=0}^{\infty }\frac{{(\pm i\alpha \sin \theta )}^{n}}{\sqrt{n!}}|n\rangle$ for $\alpha \in {\bf{R}}$. Then, we measure the qubus beam of path b using PNR measurement. If the measurement result is ${|0\rangle }^{{\rm{b}}}$ (no photon), we can obtain hybrid entanglement as $({|H\rangle }_{1}^{{\rm{A}}}{|{s}\rangle }_{1}{|V\rangle }_{2}^{{\rm{B}}}{|{l}\rangle }_{2}+{|V\rangle }_{1}^{{\rm{A}}}{|{l}\rangle }_{1}{|H\rangle }_{2}^{{\rm{B}}}{|{s}\rangle }_{2})/\sqrt{2}$. Otherwise $({|n\rangle}^{{\rm{b}}},{\rm{a}}{\rm{n}}{\rm{d}}\,n\ne 0)$, the output state is $({|V\rangle }_{1}^{{\rm{A}}}{|{l}\rangle }_{1}{|V\rangle }_{2}^{{\rm{B}}}{|{l}\rangle }_{2}+{|H\rangle }_{1}^{{\rm{A}}}{|{s}\rangle }_{1}{|H\rangle }_{2}^{{\rm{B}}}{|{s}\rangle }_{2})/\sqrt{2}$ via feed-forward (phase shifter (PS): Φ^*n*^) according to result *n*, as described in Fig. 1.

### Parity gate (PG) using XKNLs, qubus beams, and PNR measurement

In a strict sense, a PG is almost an HEG without time-bin encodings. A PG consists of four PBSs, conditional phase shifts θ (four XKNLs: only positive phase) in Kerr media, two linear phase shifters (−θ), and two BSs in qubus beams without a difference in the length of the optical paths (no time-bin encoding), as shown in Fig. 1. After the interactions of the input state (${|R\rangle }_{1}^{{\rm{A}}}{|R\rangle }_{2}^{{\rm{B}}}\otimes {|\alpha \rangle }^{{\rm{a}}}$) in the PG, the output state is given by

2 $$\begin{array}{cc}|{{\boldsymbol{\psi }}}_{{\rm{P}}{\rm{G}}}\rangle & \,=\,{|\alpha \rangle}^{{\rm{a}}}\otimes \frac{1}{\sqrt{2}}[\frac{1}{\sqrt{2}}({|H\rangle}_{1}^{{\rm{A}}}{|V\rangle}_{2}^{{\rm{B}}}+{|V\rangle}_{1}^{{\rm{A}}}{|H\rangle}_{2}^{{\rm{B}}})]\otimes {|0\rangle}^{{\rm{b}}}\\  & \quad +\,{|\alpha \cos \theta \rangle}^{{\rm{a}}}\otimes \frac{1}{\sqrt{2}}\cdot {e}^{-{(\alpha \sin \theta )}^{2}/2}\sum _{n=0}^{{\rm{\infty }}}\frac{{(i\alpha \sin \theta )}^{n}}{\sqrt{n!}}[\frac{1}{\sqrt{2}}({|V\rangle}_{1}^{{\rm{A}}}{|V\rangle}_{2}^{{\rm{B}}}+{(-1)}^{n}{|H\rangle}_{1}^{{\rm{A}}}{|H\rangle}_{2}^{{\rm{B}}})]\otimes {|n\rangle}^{{\rm{b}}}.\end{array}$$

Subsequently, if the measurement result is ${|0\rangle }^{{\rm{b}}}$ (dark count) of path b in qubus beams, the output state is $({|H\rangle }_{1}^{{\rm{A}}}{|V\rangle }_{2}^{{\rm{B}}}+{|V\rangle }_{1}^{{\rm{A}}}{|H\rangle }_{2}^{{\rm{B}}})/\sqrt{2}$ (the entangled state of a single DOF). On the other hand, if ${|n\rangle}^{{\rm{b}}}\,{\rm{a}}{\rm{n}}{\rm{d}}\,\because n\ne 0$, we can obtain $({|V\rangle }_{1}^{{\rm{A}}}{|V\rangle }_{2}^{{\rm{B}}}+{|H\rangle }_{1}^{{\rm{A}}}{|H\rangle }_{2}^{{\rm{B}}})/\sqrt{2}$ using feed-forward (PS Φ^*n*^) according to result *n*, as described in Fig. 1.

### Three time-bin encoders (T1, T2, and T3) using circularly PBSs (CPBSs), which transmit $|{\boldsymbol{R}}\rangle$ and reflect $|{\boldsymbol{L}}\rangle$, with delay-line (DL) or PBSs

For the encoding time-bin with regard to the types of polarization, we can simply use the linear optical devices (PBSs and CPBSs) and the different lengths of the path in optical fiber, as follows:

3 $${|H\rangle}_{1}^{{\rm{A}}}\mathop{\Rightarrow }\limits^{{\rm{T}}1}\frac{1}{\sqrt{2}}({|R\rangle}_{1}^{{\rm{A}}}{|s\rangle}_{1}+{|L\rangle}_{1}^{{\rm{A}}}{|l\rangle}_{1}),\,{|H\rangle}_{1}^{{\rm{A}}}\mathop{\Rightarrow }\limits^{{\rm{T}}2}\frac{1}{\sqrt{2}}({|R\rangle}_{1}^{{\rm{A}}}{|l\rangle}_{1}+{|L\rangle}_{1}^{{\rm{A}}}{|s\rangle}_{1}),\,{|R\rangle }_{1}^{{\rm{A}}}\mathop{\Rightarrow }\limits^{{\rm{T}}3}\frac{1}{\sqrt{2}}({|H\rangle }_{1}^{{\rm{A}}}{|s\rangle }_{1}+{|V\rangle }_{1}^{{\rm{A}}}{|l\rangle }_{1}),$$

where DL in T2 plays a role as the long length of the path for time delay (interval) *l*.

**Figure 2:**
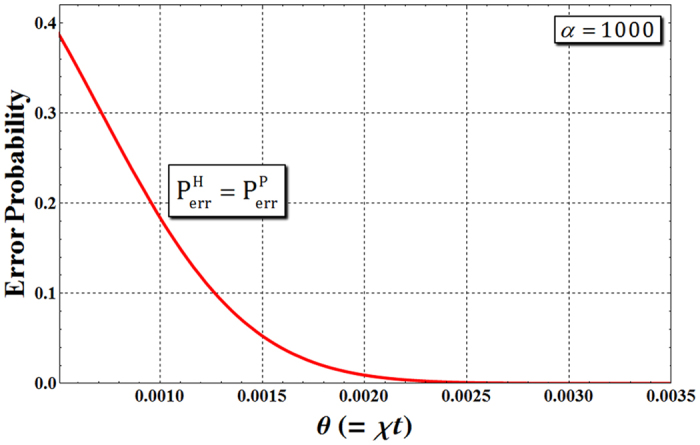
The error probabilities of an HEG (${{\rm{P}}}_{{\rm{err}}}^{{\rm{H}}}$) and a PG (${{\rm{P}}}_{{\rm{err}}}^{{\rm{P}}}$) using XKNLs, qubus beams, and PNR measurement when the amplitude of the probe beam *α* = 1000. The red line is ${{\rm{P}}}_{{\rm{err}}}^{{\rm{H}}}={{\rm{P}}}_{{\rm{err}}}^{{\rm{P}}}\approx {e}^{-{\alpha }^{2}{\theta }^{2}}/2$ in Eq. 4.

Now, we analyze the error probabilities for reliable performance in the ideal case (no decoherence effect). Let us assume that we measure the qubus beams of path b, Eq. 1, of output state through HEG using PNR measurement. If the measurement result is ${|0\rangle }^{{\rm{b}}}$ (no photon), the output state is considered as $({|H\rangle }_{1}^{{\rm{A}}}{|{s}\rangle }_{1}{|V\rangle }_{2}^{{\rm{B}}}{|{l}\rangle }_{2}+{|V\rangle }_{1}^{{\rm{A}}}{|{l}\rangle }_{1}{|H\rangle }_{2}^{{\rm{B}}}{|{s}\rangle }_{2})/\sqrt{2}$. However, if the above result, ${|0\rangle }^{{\rm{b}}}$, is detected from ${|\pm i\alpha \sin \theta \rangle }^{{\rm{b}}}$, the actual output state is $({|V\rangle }_{1}^{{\rm{A}}}{|{l}\rangle }_{1}{|V\rangle }_{2}^{{\rm{B}}}{|{l}\rangle }_{2}+{(-1)}^{n}{|H\rangle }_{1}^{{\rm{A}}}{|{s}\rangle }_{1}{|H\rangle }_{2}^{{\rm{B}}}{|{s}\rangle }_{2})/\sqrt{2}$, as described in Eq. 1. Therefore, the failed output state from HEG occurs in the incorrect measurement result. Thus, the error probabilities ${{\rm{P}}}_{{\rm{err}}}^{{\rm{H}}}$ of HEG and ${{\rm{P}}}_{{\rm{err}}}^{{\rm{P}}}$ of PG, which are the probabilities to detect ${|0\rangle }^{{\rm{b}}}$ (no photon) in ${|\pm i\alpha \sin \theta \rangle }^{{\rm{b}}}$ on path b, can be calculated as

4 $${{\rm{P}}}_{{\rm{e}}{\rm{r}}{\rm{r}}}^{{\rm{H}}}={{\rm{P}}}_{{\rm{e}}{\rm{r}}{\rm{r}}}^{{\rm{P}}}=\frac{1}{2}{e}^{-{(\alpha \sin \theta )}^{2}}\approx \frac{1}{2}{e}^{-{\alpha }^{2}{\theta }^{2}}\quad \because {\sin }^{2}\theta \approx {\theta }^{2},$$

where the amplitude of the probe beam is large (*α* ≫ 1) and *θ* ≪ 1. Figure 2 shows the error probabilities of HEG and PG according to the magnitude of the XKNL (*θ* = *χt*) with fixed the amplitude of probe beam (*α*). If we take *α* = 1000, the error probabilities of the HEG and PG are ${{\rm{P}}}_{{\rm{err}}}^{{\rm{H}}}={{\rm{P}}}_{{\rm{err}}}^{{\rm{P}}} < {10}^{-3}$ for the magnitude of the XKNL at *θ* > 0.0025, as shown in Fig. 2. This means that the error probabilities ${{\rm{P}}}_{{\rm{e}}{\rm{r}}{\rm{r}}}^{{\rm{H}}},\,{{\rm{P}}}_{{\rm{e}}{\rm{r}}{\rm{r}}}^{{\rm{P}}}$ approach to zero when increasing the magnitude of XKNL (controlled phase shift) with the amplitude of probe beam. Even though the magnitude of the nonlinearity could be significantly enhanced by electromagnetically induced transparency (EIT), *θ* ≈ 10^−2^ ^57, 58^, natural XKNLs are extremely weak: *θ* ≈ 10^−18^ ^59^. Also, since the minus conditional phase shift (−θ) in the XKNL is known to be difficult to experimentally implement^60^, we propose that an HEG and a PG based on qubus beams and PNR measurement do not require a negative XKNL (−θ).

Consequently, for the generation of hybrid entanglement in two DOFs (polarization and time-bin) and entanglement in a single DOF (polarization), the proposed HEG and PG using weak XKNLs, qubus beams, and PNR measurement are efficient and feasible in the ideal case (no decoherence effect). And we will employ the HEG, PG, and time-bin encoders (T1, T2, and T3) to distribute, purify, and generate the various entanglements (entanglement channels).

## Distribution of hybrid entanglement and hyperentanglement using HEG, PG, and time-bin encoders

**Figure 3:**
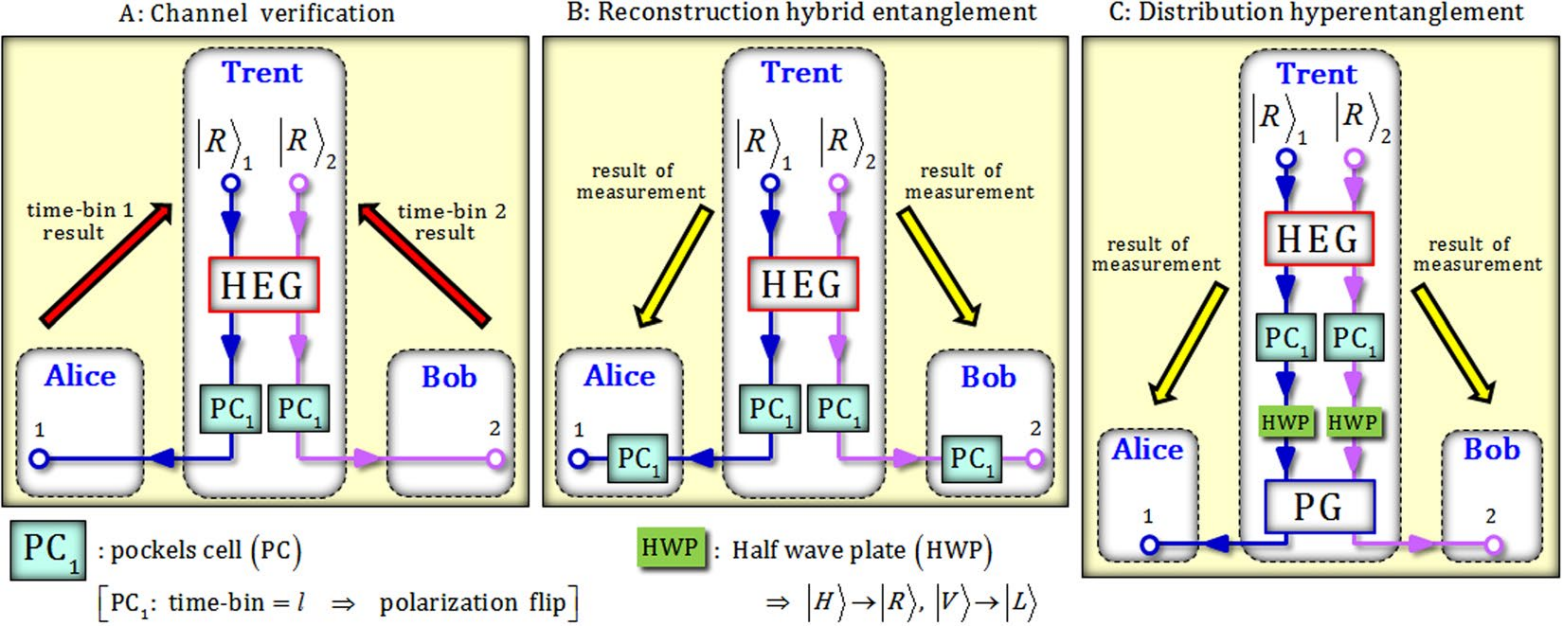
(**A**) Channel verification: Trent can distribute the state of correlation (two photons) in a time-bin using HEGs and Pockels cells (PCs). And then, the quantum channel is verified by the measurement results (time-bins) of Alice and Bob. (**B**) Reconstruction hybrid entanglement: Alice and Bob can recover the hybrid entangled state (polarization and time-bin) from the state (two photons) of correlation in a time-bin by PCs. (**C**) Distribution hyperentanglement: Trent can generate and distribute the hyperentangled state (polarization, and time-bin, respectively) via HEG, PG, PCs, and half wave plates.

We propose the distribution and generation of hybrid entanglement for two DOFs (polarization and time-bin) with verification of the channel, and distribution of hyperentanglement having correlations for two DOFs (polarization and time-bin) via HEG and PG, as shown in Fig. 1, and linear optical devices. Furthermore, we show that the entanglement in a single DOF (polarization) for two photons can be purified from correlation of the time-bin using only time-bin encoders (T1s and T2s) and linear optical devices under a noisy (bit-flip) channel, and the purified entangled state in a single DOF (polarization) can be extended to a hybrid entangled state of four photons in two DOFs (polarization and time-bin) via local operations (HEGs and T3s) and classical communications. Figure 3 shows the various schemes for the distribution and generation of hybrid entanglement and hyperentanglement, as follows.

### Scheme (A) Channel verification

First, let us assume the generating hybrid entangled state of Trent using the HEG in Fig. 1 and Eq. 1 is given by

5 $$\begin{array}{c}{|0\rangle }_{{\rm{T}}{\rm{H}}}^{{\rm{b}}}\Rightarrow |{{\boldsymbol{\Psi }}}_{{\rm{H}}{\rm{B}}}^{+}\rangle \equiv \frac{1}{\sqrt{2}}({|H\rangle }_{1}{|s\rangle }_{1}{|V\rangle }_{2}{|l\rangle }_{2}+{|V\rangle }_{1}{|l\rangle }_{1}{|H\rangle }_{2}{|s\rangle }_{2}),\\ {|n\rangle }_{{\rm{T}}{\rm{H}}}^{{\rm{b}}}\Rightarrow |{{\boldsymbol{\Phi }}}_{{\rm{H}}{\rm{B}}}^{+}\rangle \equiv \frac{1}{\sqrt{2}}({|H\rangle }_{1}{|s\rangle }_{1}{|H\rangle }_{2}{|s\rangle }_{2}+{|V\rangle }_{1}{|l\rangle }_{1}{|V\rangle }_{2}{|l\rangle }_{2}),\end{array}$$

where the hybrid entangled state ($|{{\boldsymbol{\Psi }}}_{{\rm{HB}}}^{+}\rangle$ or $|{{\boldsymbol{\Phi }}}_{{\rm{HB}}}^{+}\rangle$) for two DOFs (polarization and time-bin) of two photons is generated with Trent’s result (${|0\rangle }_{{\rm{TH}}}^{{\rm{b}}}$ or ${|n\rangle }_{{\rm{TH}}}^{{\rm{b}}}\because n\ne 0$) from PNR measurement in the HEG. And then, Trent utilizes Pockels cells (PCs), which affect a bit-flip operation on the polarization at a specific time^30, 31, 34, 61–63^, to store the correlation from two DOFs (polarization and time-bin) into only a single DOF (time-bin), as follows:

6 $$\begin{array}{c}|{{\boldsymbol{\Psi }}}_{{\rm{H}}{\rm{B}}}^{+}\rangle \mathop{\Rightarrow }\limits^{{{\rm{P}}{\rm{C}}}_{1}}{|H\rangle }_{1}{|H\rangle }_{2}\otimes \frac{1}{\sqrt{2}}({|s\rangle }_{1}{|l\rangle }_{2}+{|l\rangle }_{1}{|s\rangle }_{2}),\\ |{{\boldsymbol{\Phi }}}_{{\rm{H}}{\rm{B}}}^{+}\rangle \mathop{\Rightarrow }\limits^{{{\rm{P}}{\rm{C}}}_{1}}{|H\rangle }_{1}{|H\rangle }_{2}\otimes \frac{1}{\sqrt{2}}({|s\rangle }_{1}{|s\rangle }_{2}+{|l\rangle }_{1}{|l\rangle }_{2}),\end{array}$$

where the action of PC_1_ flips the polarization of the photon at time-bin $|{l}\rangle$. Subsequently, two photons are transmitted to users (Alice: photon 1, and Bob: photon 2). After the users measure the polarization and time-bin of their own photons, they announce the information of the time-bins ($|{s}\rangle$ or $|{l}\rangle$) to Trent, as shown in Fig. 3A. Consequently, Trent can confirm the security of the quantum channel in accordance with the received information of the time-bins (from the users) and the result of PNR measurement in the HEG (${|0\rangle }_{{\rm{T}}{\rm{H}}}^{{\rm{b}}}:{|s\rangle }_{1}{|l\rangle }_{2}\,{\rm{o}}{\rm{r}}\,{|l\rangle }_{1}{|s\rangle }_{2}$ and ${|n\rangle }_{{\rm{T}}{\rm{H}}}^{{\rm{b}}}:{|s\rangle }_{1}{|s\rangle }_{2}\,{\rm{o}}{\rm{r}}\,{|l\rangle }_{1}{|l\rangle }_{2}$).

### Scheme (B) Reconstruction hybrid entanglement

After the process of verification, Alice and Bob can recover hybrid entangled state ($|{{\boldsymbol{\Psi }}}_{{\rm{HB}}}^{+}\rangle$ or $|{{\boldsymbol{\Phi }}}_{{\rm{HB}}}^{+}\rangle$ in Eq. 5) from the stored correlation in the time-bin (two photons), Eq. 6, using only PC_1_s in other sessions. Subsequently, Alice and Bob can share hybrid entanglement ($|{{\boldsymbol{\Psi }}}_{{\rm{HB}}}^{+}\rangle$ or $|{{\boldsymbol{\Phi }}}_{{\rm{HB}}}^{+}\rangle$) through Trent’s results from PNR measurement in the HEG.

### Scheme (C) Distribution hyperentanglement

By modification (attaching the PG and half wave plates [HWPs]) of scheme (A), we can acquire the generation of hyperentanglement having its own correlations for two DOFs (polarization and time-bin). After an HEG and PC_1_s, the state of the stored correlation in the time-bin (two photons) is given as Eq. 6. Then, two photons that pass HWPs and a PG will be transformed according to the results of PNR measurement in the HEG and the PG, as follows:

7 $$\begin{array}{c}\{{|0\rangle }_{{\rm{TH}}}^{{\rm{b}}}{\rm{and}}{|0\rangle }_{{\rm{TP}}}^{{\rm{b}}}\}\Rightarrow ({|H\rangle }_{1}{|V\rangle }_{2}+{|V\rangle }_{1}{|H\rangle }_{2})/\sqrt{2}\otimes ({|{s}\rangle }_{1}{|{l}\rangle }_{2}+{|{l}\rangle }_{1}{|{s}\rangle }_{2})/\sqrt{2},\\ \{{|0\rangle }_{{\rm{TH}}}^{{\rm{b}}}{\rm{and}}{|j\rangle }_{{\rm{TP}}}^{{\rm{b}}}\}\Rightarrow ({|H\rangle }_{1}{|H\rangle }_{2}+{|V\rangle }_{1}{|V\rangle }_{2})/\sqrt{2}\otimes ({|{s}\rangle }_{1}{|{l}\rangle }_{2}+{|{l}\rangle }_{1}{|{s}\rangle }_{2})/\sqrt{2},\\ \{{|n\rangle }_{{\rm{TH}}}^{{\rm{b}}}{\rm{and}}{|0\rangle }_{{\rm{TP}}}^{{\rm{b}}}\}\Rightarrow ({|H\rangle }_{1}{|V\rangle }_{2}+{|V\rangle }_{1}{|H\rangle }_{2})/\sqrt{2}\otimes ({|{s}\rangle }_{1}{|{s}\rangle }_{2}+{|{l}\rangle }_{1}{|{l}\rangle }_{2})/\sqrt{2},\\ \{{|n\rangle }_{{\rm{TH}}}^{{\rm{b}}}{\rm{and}}{|j\rangle }_{{\rm{TP}}}^{{\rm{b}}}\}\Rightarrow ({|H\rangle }_{1}{|H\rangle }_{2}+{|V\rangle }_{1}{|V\rangle }_{2})/\sqrt{2}\otimes ({|{s}\rangle }_{1}{|{s}\rangle }_{2}+{|{l}\rangle }_{1}{|{l}\rangle }_{2})/\sqrt{2},\end{array}$$

where ${|0\rangle }_{{\rm{T}}{\rm{H}}}^{{\rm{b}}}\,({|n\rangle }_{{\rm{T}}{\rm{H}}}^{{\rm{b}}})$ and ${|0\rangle }_{{\rm{T}}{\rm{P}}}^{{\rm{b}}}\,({|j\rangle }_{{\rm{T}}{\rm{P}}}^{{\rm{b}}}\,\because j\ne 0)$ are the results of PNR measurement of the HEG and PG. Consequently, the hyperentanglement having its own correlations for two DOFs (polarization and time-bin) can be shared through Trent’s results from PNR measurement in the HEG and PG after Alice and Bob receive two photons (1 and 2), respectively.

Now, we present the schemes for the purification of the entangled state (two photons) for a single DOF (polarization)^31^ from the state-stored (two photons) correlation in the time-bin, as seen in Eq. 6 by the time-bin encoders and linear optical devices (including PCs). We also extend the hybrid entangled state of four photons for two DOFs (polarization and time-bin) from the purified entangled state using local operations (nonlinear: HEGs; linear: PCs and T3s) and classical communications under a noisy (bit-flip) channel.

### Scheme (D) Purification entanglement under noise

In practice, the purification scheme should be positively necessary to establish the reliable construction of an entanglement channel since the noise in the quantum channel for the transmission of photons is unavoidable. Thus, let us assume that the bit-flip (exactly polarization-flip) noise against the transferring two photons occurs between Trent and the users^31^. On Trent’s side, via HEG and PC_1_s, the generated state is given as Eq. 6, and this means that the correlation (or anti-correlation) of the formal hybrid entangled state (polarization and time-bin) can be stored in only a single DOF (time-bin). Subsequently, due to the noise (bit-flip), the corrupt state of two photons is expressed as

8 $$\begin{array}{cc}{|H\rangle }_{1}{|H\rangle }_{2}\otimes \frac{1}{\sqrt{2}}({|s\rangle }_{1}{|l\rangle }_{2}+{|l\rangle }_{1}{|s\rangle }_{2})\mathop{\to }\limits^{{\rm{N}}{\rm{o}}{\rm{i}}{\rm{s}}{\rm{y}}}|{{\boldsymbol{\Psi }}}_{{\rm{B}}{\rm{F}}}^{+}\rangle \,\equiv \, & ({\alpha }_{1}{|H\rangle }_{1}+{\beta }_{1}{|V\rangle }_{1})({\alpha }_{2}{|H\rangle }_{2}+{\beta }_{2}{|V\rangle }_{2})\\  & \otimes \frac{1}{\sqrt{2}}({|s\rangle }_{1}{|l\rangle }_{2}+{|l\rangle }_{1}{|s\rangle }_{2}),\\ {|H\rangle }_{1}{|H\rangle }_{2}\otimes \frac{1}{\sqrt{2}}({|s\rangle }_{1}{|s\rangle }_{2}+{|l\rangle }_{1}{|l\rangle }_{2})\mathop{\to }\limits^{{\rm{N}}{\rm{o}}{\rm{i}}{\rm{s}}{\rm{y}}}|{{\boldsymbol{\Phi }}}_{{\rm{B}}{\rm{F}}}^{+}\rangle \,\equiv \, & ({\alpha }_{1}{|H\rangle }_{1}+{\beta }_{1}{|V\rangle }_{1})({\alpha }_{2}{|H\rangle }_{2}+{\beta }_{2}{|V\rangle }_{2})\\  & \otimes \frac{1}{\sqrt{2}}({|s\rangle }_{1}{|s\rangle }_{2}+{|l\rangle }_{1}{|l\rangle }_{2}),\end{array}$$

where the occurrence of bit-flip noise brings the result, $|H\rangle \to \alpha |H\rangle +\beta |V\rangle$, with respect to the polarization in photons when the correlation is stored in the time-bin as $|{{\boldsymbol{\Psi }}}_{{\rm{BF}}}^{+}\rangle$ and $|{{\boldsymbol{\Phi }}}_{{\rm{BF}}}^{+}\rangle$. And then, Alice and Bob perform the process of purification consisting of time-bin encoders (T1 and T2), ${{\rm{PC}}}_{{\rm{2}}}{\rm{s}}$, and HWPs. Then, the purified entangled state for a single DOF (polarization), $|{{\rm{P}}}^{-}\rangle$ and $|{{\rm{P}}}^{+}\rangle$, is given by

9 $$\begin{array}{c}|{{\boldsymbol{\Psi }}}_{{\rm{B}}{\rm{F}}}^{+}\rangle \mathop{\Rightarrow }\limits^{{\rm{p}}{\rm{u}}{\rm{r}}{\rm{i}}{\rm{f}}{\rm{y}}}|{{\rm{P}}}^{-}\rangle =\frac{1}{\sqrt{2}}({|R\rangle }_{1}{|L\rangle }_{2}+{|L\rangle }_{1}{|R\rangle }_{2})\otimes ({\alpha }_{1}{|{l}^{2}\rangle }_{1}+{\beta }_{1}{|{s}^{2}\rangle }_{1})({\alpha }_{2}{|{l}^{2}\rangle }_{2}+{\beta }_{2}{|{s}^{2}\rangle }_{2}),\\ |{{\boldsymbol{\Phi }}}_{{\rm{B}}{\rm{F}}}^{+}\rangle \mathop{\Rightarrow }\limits^{{\rm{p}}{\rm{u}}{\rm{r}}{\rm{i}}{\rm{f}}{\rm{y}}}|{{\rm{P}}}^{+}\rangle =\frac{1}{\sqrt{2}}({|R\rangle }_{1}{|R\rangle }_{2}+{|L\rangle }_{1}{|L\rangle }_{2})\otimes ({\alpha }_{1}{|{l}^{2}\rangle }_{1}+{\beta }_{1}{|{s}^{2}\rangle }_{1})({\alpha }_{2}{|{l}^{2}\rangle }_{2}+{\beta }_{2}{|{s}^{2}\rangle }_{2}),\end{array}$$

where the action of PC_2_ flips the photon polarization at time-bins $|{sl}\rangle$ and $|{ls}\rangle$. We define the time-bin time intervals as $|{s}^{2}\rangle \equiv |ssl\,{\rm{o}}{\rm{r}}\,sls\,{\rm{o}}{\rm{r}}\,lss\rangle$ and $|{l}^{2}\rangle \equiv |lls\,{\rm{o}}{\rm{r}}\,lsl\,{\rm{o}}{\rm{r}}\,sll\rangle$. Consequently, Alice and Bob can share the entangled state ($|{{\rm{P}}}^{-}\rangle$ or $|{{\rm{P}}}^{+}\rangle$) for a single DOF (polarization), under a noisy (bit-flip) channel, through the purifications (using only linear optical devices) and the result of PNR measurement of the HEG.

### Scheme (E) Generation of four-photon hybrid entanglement

**Figure 4:**
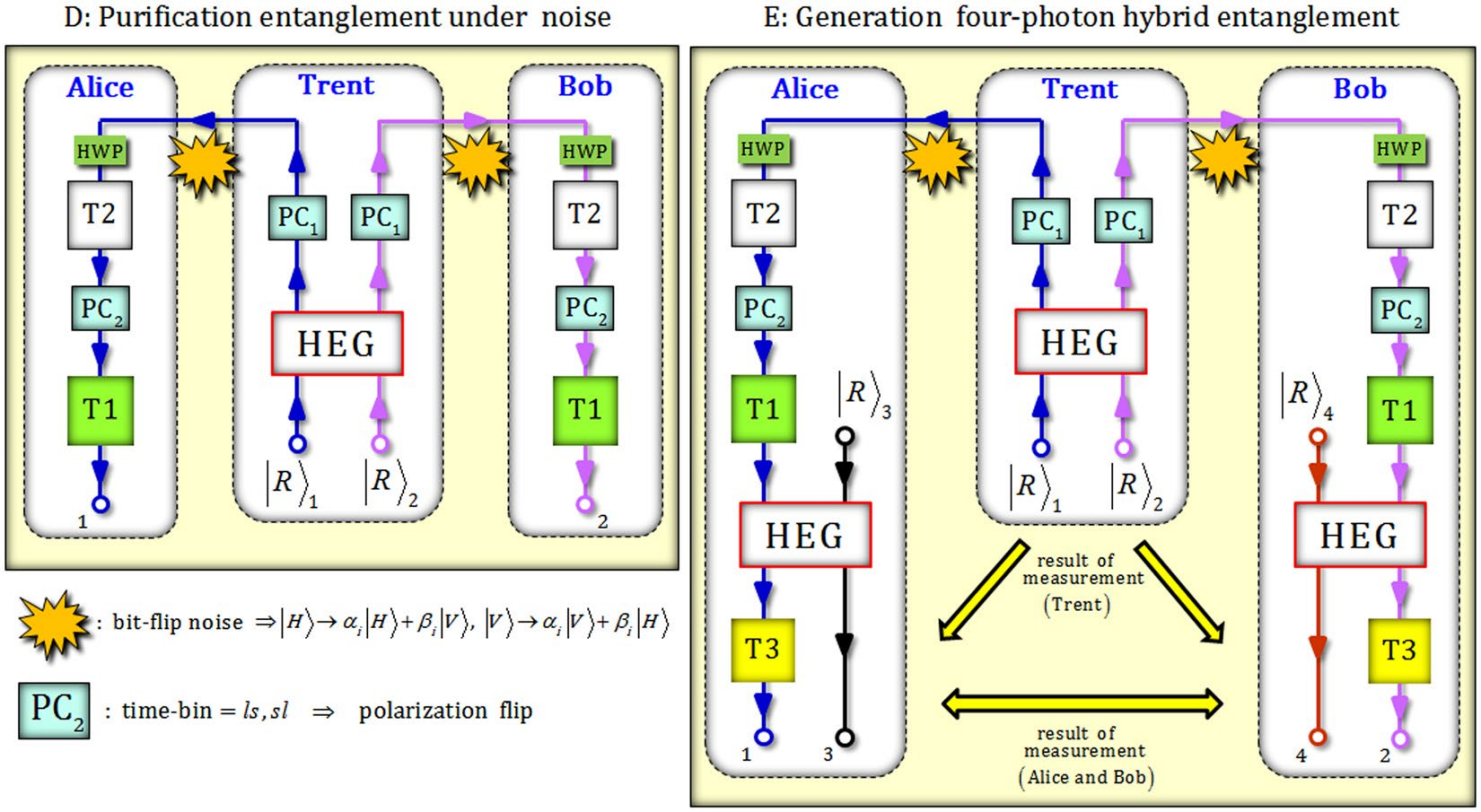
(**D**) Purification entanglement under noise: After Trent generates the state of the stored correlation in the time-bin (two photons), as seen in Eq. 6, by the application of PC_1_s to the hybrid entangled state (polarization and time-bin), two photons (1 and 2) are transferred to Alice and Bob under a noisy (bit-flip) channel. Alice and Bob can purify the entangled state in only a single DOF (polarization) from the impaired hybrid entangled state under bit-flip noise using time-bin encoders, PCs, and linear optical devices. (**E**) Generation of four-photon hybrid entanglement: The purified entangled state (polarization) through scheme (**D**) can be extended to a hybrid entangled state (four photons) in two DOFs (polarization and time-bin) using two HEGs (nonlinearity), and two time-bin encoders (T3).

For expandable entanglement, we can generate a hybrid entangled state (four photons) for two DOFs (polarization and time-bin) from the resulting state of scheme (D) in Eq. 9 by utilizing HEGs (nonlinearity) and time-bin encoders (T3s). Alice and Bob prepare two photons ${|R\rangle }_{3}$ and ${|R\rangle }_{4}$, respectively. Then, they perform the process of HEGs (including feed-forwards due to PNR measurement, as seen in Eq. 1) and T3s as described in Fig. 4. If we consider the resulting state from scheme (D) as $|{{\rm{P}}}^{-}\rangle$, which was decided by the result (${|0\rangle }_{{\rm{TH}}}^{{\rm{b}}}$) of PNR measurement in the HEG on Trent’s side, as shown in Eq. 5, the final state (a four-photon hybrid entangled state) will be given according to the results of PNR measurement in the HEGs (Alice and Bob), as follows: (appendix)

10 $$\begin{array}{cc}\{{|0\rangle }_{{\rm{A}}{\rm{H}}}^{{\rm{b}}}\,{\rm{a}}{\rm{n}}{\rm{d}}\,{|0\rangle }_{{\rm{B}}{\rm{H}}}^{{\rm{b}}}\}\quad \\ \Rightarrow {|{{\rm{F}}}_{1}^{-}\rangle }_{1324}\equiv \frac{1}{\sqrt{2}} & [{|H\rangle }_{1}({\alpha }_{1}{|{s}^{3}\rangle }_{1}+{\beta }_{1}{|{s}^{4}\rangle }_{1}){|V\rangle }_{3}{|l\rangle }_{3}{|H\rangle }_{2}({\alpha }_{2}{|{s}^{3}\rangle }_{2}+{\beta }_{2}{|{s}^{4}\rangle }_{2}){|V\rangle }_{4}{|l\rangle }_{4}\\  & -{|V\rangle }_{1}({\alpha }_{1}{|{l}^{4}\rangle }_{1}+{\beta }_{1}{|{l}^{3}\rangle }_{1}){|H\rangle }_{3}{|s\rangle }_{3}{|V\rangle }_{2}({\alpha }_{2}{|{l}^{4}\rangle }_{2}+{\beta }_{2}{|{l}^{3}\rangle }_{2}){|H\rangle }_{4}{|s\rangle }_{4}],\\ \{{|0\rangle }_{{\rm{A}}{\rm{H}}}^{{\rm{b}}}\,{\rm{a}}{\rm{n}}{\rm{d}}\,{|k\rangle }_{{\rm{B}}{\rm{H}}}^{{\rm{b}}}\}\quad \\ \Rightarrow {|{{\rm{F}}}_{2}^{-}\rangle }_{1324}\equiv \frac{1}{\sqrt{2}} & [{|H\rangle }_{1}({\alpha }_{1}{|{s}^{3}\rangle }_{1}+{\beta }_{1}{|{s}^{4}\rangle }_{1}){|V\rangle }_{3}{|l\rangle }_{3}{|H\rangle }_{2}({\alpha }_{2}{|{s}^{3}\rangle }_{2}+{\beta }_{2}{|{s}^{4}\rangle }_{2}){|H\rangle }_{4}{|s\rangle }_{4}\\  & -{|V\rangle }_{1}({\alpha }_{1}{|{l}^{4}\rangle }_{1}+{\beta }_{1}{|{l}^{3}\rangle }_{1}){|H\rangle }_{3}{|s\rangle }_{3}{|V\rangle }_{2}({\alpha }_{2}{|{l}^{4}\rangle }_{2}+{\beta }_{2}{|{l}^{3}\rangle }_{2}){|H\rangle }_{4}{|l\rangle }_{4}],\\ \{{|m\rangle }_{{\rm{A}}{\rm{H}}}^{{\rm{b}}}\,{\rm{a}}{\rm{n}}{\rm{d}}\,{|0\rangle }_{{\rm{B}}{\rm{H}}}^{{\rm{b}}}\}\quad \\ \Rightarrow {|{{\rm{F}}}_{3}^{-}\rangle }_{1324}\equiv \frac{1}{\sqrt{2}} & [{|H\rangle }_{1}({\alpha }_{1}{|{s}^{3}\rangle }_{1}+{\beta }_{1}{|{s}^{4}\rangle }_{1}){|H\rangle }_{3}{|s\rangle }_{3}{|H\rangle }_{2}({\alpha }_{2}{|{s}^{3}\rangle }_{2}+{\beta }_{2}{|{s}^{4}\rangle }_{2}){|V\rangle }_{4}{|l\rangle }_{4}\\  & -{|V\rangle }_{1}({\alpha }_{1}{|{l}^{4}\rangle }_{1}+{\beta }_{1}{|{l}^{3}\rangle }_{1}){|V\rangle }_{3}{|l\rangle }_{3}{|V\rangle }_{2}({\alpha }_{2}{|{l}^{4}\rangle }_{2}+{\beta }_{2}{|{l}^{3}\rangle }_{2}){|H\rangle }_{4}{|s\rangle }_{4}],\\ \{{|m\rangle }_{{\rm{A}}{\rm{H}}}^{{\rm{b}}}\,{\rm{a}}{\rm{n}}{\rm{d}}\,{|k\rangle }_{{\rm{B}}{\rm{H}}}^{{\rm{b}}}\}\quad \\ \Rightarrow {|{{\rm{F}}}_{4}^{-}\rangle }_{1324}\equiv \frac{1}{\sqrt{2}} & [{|H\rangle }_{1}({\alpha }_{1}{|{s}^{3}\rangle }_{1}+{\beta }_{1}{|{s}^{4}\rangle }_{1}){|H\rangle }_{3}{|s\rangle }_{3}{|H\rangle }_{2}({\alpha }_{2}{|{s}^{3}\rangle }_{2}+{\beta }_{2}{|{s}^{4}\rangle }_{2}){|H\rangle }_{4}{|s\rangle }_{4}\\  & -{|V\rangle }_{1}({\alpha }_{1}{|{l}^{4}\rangle }_{1}+{\beta }_{1}{|{l}^{3}\rangle }_{1}){|V\rangle }_{3}{|l\rangle }_{3}{|V\rangle }_{2}({\alpha }_{2}{|{l}^{4}\rangle }_{2}+{\beta }_{2}{|{l}^{3}\rangle }_{2}){|V\rangle }_{4}{|l\rangle }_{4}],\end{array}$$

where ${|0\rangle }_{{\rm{AH}}}^{{\rm{b}}}({|m\rangle }_{{\rm{AH}}}^{{\rm{b}}}\because m\ne 0)$ and ${|0\rangle }_{{\rm{BH}}}^{{\rm{b}}}({|k\rangle }_{{\rm{BH}}}^{{\rm{b}}}\because k\ne 0)$ are the results of PNR measurement of the HEGs on Alice’s and Bob’s sides. We define the time-bins as $|{s}^{4}\rangle \equiv |lssss\,{\rm{o}}{\rm{r}}......{\rm{o}}{\rm{r}}\,ssssl\rangle$, $|{s}^{3}\rangle \equiv |llsss\,{\rm{o}}{\rm{r}}......{\rm{o}}{\rm{r}}\,sssll\rangle$ (short interval), and $|{l}^{4}\rangle \equiv |lllls\,{\rm{o}}{\rm{r}}......{\rm{o}}{\rm{r}}\,sllll\rangle$, $|{l}^{3}\rangle \equiv |lllss\,{\rm{o}}{\rm{r}}......{\rm{o}}{\rm{r}}\,sslll\rangle$ (long interval). Thus, we can consider the time-bins, ${\alpha }_{1}{|{{s}}^{{\rm{3}}}\rangle }_{1}+{\beta }_{1}{|{{s}}^{{\rm{4}}}\rangle }_{1}$ and ${\alpha }_{2}{|{{l}}^{{\rm{4}}}\rangle }_{2}+{\beta }_{2}{|{{l}}^{{\rm{3}}}\rangle }_{2}$, as the short intervals ($|{s}^{3}\rangle ,|{s}^{4}\rangle$) and the long intervals $|{{l}}^{{\rm{3}}}\rangle ,|{{l}}^{{\rm{4}}}\rangle$. Table 1 shows the final hybrid entangled state (four photons) for two DOFs (polarization and time-bin) in accordance with the results of PNR measurement in the HEGs of Trent, (${|0\rangle }_{{\rm{TH}}}^{{\rm{b}}}$ or ${|n\rangle }_{{\rm{TH}}}^{{\rm{b}}}$), Alice (${|0\rangle }_{{\rm{AH}}}^{{\rm{b}}}$ or ${|m\rangle }_{{\rm{AH}}}^{{\rm{b}}}$), and Bob (${|0\rangle }_{{\rm{BH}}}^{{\rm{b}}}$ or ${|k\rangle }_{{\rm{BH}}}^{{\rm{b}}}$). Here ${|{{\rm{F}}}_{{\rm{i}}}^{+}\rangle }_{1324}$ is defined as the flipped relative phase in ${|{{\rm{F}}}_{{\rm{i}}}^{-}\rangle }_{1324}$ of Eq. 10. Consequently, Alice and Bob can share hybrid entanglement (four photons) for two DOFs through the announcement of the results of PNR measurement in the HEGs of Trent and the users.

**Table 1:** According to the results of PNR measurement in the HEGs of Trent, Alice, and Bob, the distributed hybrid entanglement (four photons) for two DOFs (polarization and time-bin) between Alice and Bob.

Trent’s result from PNR measurement	Alice’s result from PNR measurement	Bob’s result from PNR measurement	Distributed hybrid entangled state (four photons)
$$\begin{array}{c}{|0\rangle }_{{\rm{TH}}}^{{\rm{b}}}\equiv {|{{\rm{F}}}_{{\rm{i}}}^{-}\rangle }_{1324}\\ {\rm{or}}\\ {|n\rangle }_{{\rm{TH}}}^{{\rm{b}}}\equiv {|{{\rm{F}}}_{{\rm{i}}}^{+}\rangle }_{1324}\end{array}$$	${|0\rangle }_{{\rm{AH}}}^{{\rm{b}}}$	${|0\rangle }_{{\rm{BH}}}^{{\rm{b}}}$	${|{{\rm{F}}}_{1}^{\pm }\rangle }_{1324}$
	${|0\rangle }_{{\rm{AH}}}^{{\rm{b}}}$	${|k\rangle }_{{\rm{BH}}}^{{\rm{b}}}$	${|{{\rm{F}}}_{2}^{\pm }\rangle }_{1324}$
	${|m\rangle }_{{\rm{AH}}}^{{\rm{b}}}$	${|0\rangle }_{{\rm{BH}}}^{{\rm{b}}}$	${|{{\rm{F}}}_{3}^{\pm }\rangle }_{1324}$
	${|m\rangle }_{{\rm{AH}}}^{{\rm{b}}}$	${|k\rangle }_{{\rm{BH}}}^{{\rm{b}}}$	${|{{\rm{F}}}_{4}^{\pm }\rangle }_{1324}$

So far, we designed the schemes to generate and distribute hybrid entanglement and hyperentanglement (schemes A through C), and also to extend to hybrid entanglement (four photons) via the process of purification (schemes D and E) using nonlinearity (weak XKNLs), time-bin encoders, and linear optical devices under a noisy channel. In our schemes, the critical components are HEGs and PGs employing XKNLs, qubus beams, and PNR measurement. But the decoherence effect, which evolves quantum pure states into a mixed state by photon loss and dephasing, occurs in these gates (HEG and PG) when experimentally implemented by our schemes (A through E) in practical optical fiber^64, 65^. Therefore, in the next section, we will demonstrate through detailed analysis that HEGs and PGs using XKNLs, qubus beams, and PNR measurement are robust against the decoherence effect^41, 42, 52^.

## Analysis of multi-photon gates using XKNLs, qubus beams, and PNR measurement for generating entanglement under the decoherence effect

In our HEG and PG, the components for analysis of the decoherence effect are photon loss of the probe (qubus) beam and dephasing coherent parameters of the photon-probe system in optical fiber^41, 42, 52, 64, 65^. The photon loss will increase error probabilities ${{\rm{P}}}_{{\rm{err}}}^{{\rm{H}}}$ (HEG) and ${{\rm{P}}}_{{\rm{err}}}^{{\rm{P}}}$ (PG) of Eq. 4, and the fidelities of the output state between the ideal case and the practical case will also decrease from evolving into a mixed state by dephasing^41, 42, 52^. Thus, we analyzed the decoherence effect of our multi-photon gates (HEG and PG) in the Kerr medium using a master equation^66^:

11 $$\frac{{\rm{\partial }}\rho }{{\rm{\partial }}t}=\hat{J}\rho +\hat{L}\rho ,\hat{J}\rho =\gamma a\rho {a}^{+},\hat{L}\rho =-\frac{\gamma }{2}({a}^{+}a\rho +\rho {a}^{+}a),$$

where *γ* is the energy decay rate. *t*(*θ*/*χ*) is the interaction time for the solution $\rho (t)=\exp [(\hat{J}+\hat{L})t]\rho (0)$. Thus, we can obtain a photon decay rate of ${\Lambda }_{t}={e}^{-\gamma t/2}$, $|{\Lambda }_{t}\alpha \rangle$ after the probe beam, $|\alpha \rangle$, passes through the Kerr medium. For good approximation of the analysis of the HEG and PG (as described elsewhere^41, 42, 52^), we can divide the whole interaction time *t* of ${\tilde{D}}_{t}$ (decoherence) and ${\tilde{X}}_{t}$ (XKNL) so it is arbitrarily small, Δ*t*(=*t*/*N*), where *N* = 10^3^, and $\theta =\chi t=\chi N{\rm{\Delta }}t=N{\rm{\Delta }}\theta$. Therefore, we can quantify the interaction of XKNLs, ${\tilde{X}}_{t}$, (simultaneously occurring with decoherence effect, ${\tilde{D}}_{t}$: photon loss and dephasing) by the process of ${\tilde{D}}_{t}{\tilde{X}}_{t}={({\tilde{D}}_{{\rm{\Delta }}t}{\tilde{X}}_{{\rm{\Delta }}t})}^{N}$ for *t*(NΔ*t*), as follows^41, 42, 52^:

12 $${({\tilde{D}}_{{\rm{\Delta }}t}{\tilde{X}}_{{\rm{\Delta }}t})}^{N}|H\rangle \langle V|\otimes |\alpha \rangle \langle \alpha |=\exp [-{\alpha }^{2}(1-{e}^{-\gamma {\rm{\Delta }}t}){\sum }_{n=1}^{N}{e}^{-\gamma {\rm{\Delta }}t(n-1)}(1-{e}^{in{\rm{\Delta }}\theta })]|H\rangle \langle V|\otimes |{\Lambda }_{t}\alpha {e}^{i\theta }\rangle \langle {\Lambda }_{t}\alpha |,$$

where ${\Lambda }_{t}={e}^{-\gamma t/2}$, *θ* = *N*Δ*θ*, and *N* = 10^3^ for *α* ∈ **R**. Here, we assume the interaction of XKNLs (the conditional phase shift) is ${\tilde{X}}_{{\rm{\Delta }}t}(|H\rangle \langle V|\otimes |\alpha \rangle \langle \alpha |)=|H\rangle \langle V|\otimes |\alpha {e}^{i\Delta \theta }\rangle \langle \alpha |$, and the decoherence effect (dephasing and photon loss) is ${\tilde{D}}_{{\rm{\Delta }}t}(|\alpha \rangle \langle \beta |)=\exp [-(1-{e}^{-\gamma \Delta t})\{-\alpha {\beta }^{\ast }+({|\alpha |}^{2}+{|\beta |}^{2})/2\}]|{\Lambda }_{{\rm{\Delta }}t}\alpha \rangle \langle {\Lambda }_{{\rm{\Delta }}t}\beta |$ from the solution of Eq. 11 ^41, 42, 49^. In experimental implementation of the multi-photon gates (HEG and PG), optical fiber of about 3000 km is required for phase shift *θ* = *π* of the XKNL^64, 65^. Thus, the amplitude (coherent state) will be reduced at a rate of *e* ^*−γ*t^, while *θ* = *π* is acquired for the 3000 km, depending on *χ*/*γ* = 0.0125 (0.364 dB/km: signal loss) of commercial fibers^64, 65^, and *χ*/*γ* = 0.0303 (0.15 dB/km, which can be obtained using pure silica core fibers^65^ in the current technology.

Now, for the feasibility of our schemes (A through E) in Section 3, we analyzed the multi-photon gate (PG) in Section 2 by modeling it as shown in Eq. 12 under the decoherence effect^41, 42, 52^. Due to the decoherence effect, the photon loss and dephasing coherent parameters should be simultaneously considered in our analysis when the PG is realized in optical fiber. Thus, output state $|{{\boldsymbol{\psi }}}_{{\rm{PG}}}\rangle$ in Eq. 2 of the PG using XKNL, qubus beams, and PNR measurement will evolve into mixed state *ρ*_PG_, as follows:

13 $${\rho }_{{\rm{PG}}}=\frac{1}{4}(\begin{array}{cccc}1 & {|K|}^{2}{|C|}^{2} & {|L|}^{2} & {|O|}^{2}{|C|}^{2}\\ {|K|}^{2}{|C|}^{2} & 1 & {|O|}^{2}{|C|}^{2} & {|L|}^{2}\\ {|L|}^{2} & {|O|}^{2}{|C|}^{2} & 1 & {|M|}^{2}{|C|}^{2}\\ {|O|}^{2}{|C|}^{2} & {|L|}^{2} & {|M|}^{2}{|C|}^{2} & 1\end{array}),$$

where the bases of the photon-probe system are ${|{\Lambda }_{t}\alpha \rangle }^{{\rm{c}}}{|HV\rangle }_{{\rm{12}}}{|0\rangle }^{{\rm{d}}}$, ${|{\Lambda }_{t}\alpha \rangle }^{{\rm{c}}}{|VH\rangle }_{{\rm{12}}}{|0\rangle }^{{\rm{d}}}$, ${|{\Lambda }_{t}\alpha \cos \theta \rangle }^{{\rm{c}}}{|VV\rangle }_{{\rm{12}}}{|i{\Lambda }_{t}\alpha \sin \theta \rangle }^{{\rm{d}}}$, and ${|{\Lambda }_{t}\alpha \cos \theta \rangle }^{{\rm{c}}}{|HH\rangle }_{{\rm{12}}}{|-i{\Lambda }_{t}\alpha \sin \theta \rangle }^{{\rm{d}}}$ from left to right and top to bottom. The coherent parameters (C, O, L, M, and K) of the off-diagonal terms, which are calculated by the decoherence (${\tilde{D}}_{t}$) and the XKNL (${\tilde{X}}_{t}$) from Eq. 12, are given by

14 $$\begin{array}{c}\,C=\exp [-{(\alpha /\sqrt{2})}^{2}(1-{e}^{-\gamma {\rm{\Delta }}t}){\sum }_{n=1}^{N}{e}^{-\gamma {\rm{\Delta }}t(n-1)}(1-{e}^{in{\rm{\Delta }}\theta })],\\ \,O=\exp [-{(\alpha /\sqrt{2})}^{2}{e}^{-\gamma t}(1-{e}^{-\gamma {\rm{\Delta }}t})(1-{e}^{i\theta }){\sum }_{n=1}^{N}{e}^{-\gamma {\rm{\Delta }}t(n-1)}],\\ \,L=\exp [-{(\alpha /\sqrt{2})}^{2}{e}^{-\gamma t}(1-{e}^{-\gamma {\rm{\Delta }}t}){\sum }_{n=1}^{N}{e}^{-\gamma {\rm{\Delta }}t(n-1)}(1-{e}^{in{\rm{\Delta }}\theta })],\\ M=\exp [-{(\alpha /\sqrt{2})}^{2}{e}^{-\gamma t}(1-{e}^{-\gamma {\rm{\Delta }}t}){\sum }_{n=1}^{N}{e}^{-\gamma {\rm{\Delta }}t(n-1)}(1-{e}^{i\cdot (n{\rm{\Delta }}\theta +\theta )})],\\ \,K=\exp [-{(\alpha /\sqrt{2})}^{2}{e}^{-\gamma t}(1-{e}^{-\gamma {\rm{\Delta }}t}){\sum }_{n=1}^{N}{e}^{-\gamma {\rm{\Delta }}t(n-1)}(1-{e}^{-i\cdot (n{\rm{\Delta }}\theta -\theta )})].\end{array}$$

**Figure 5:**
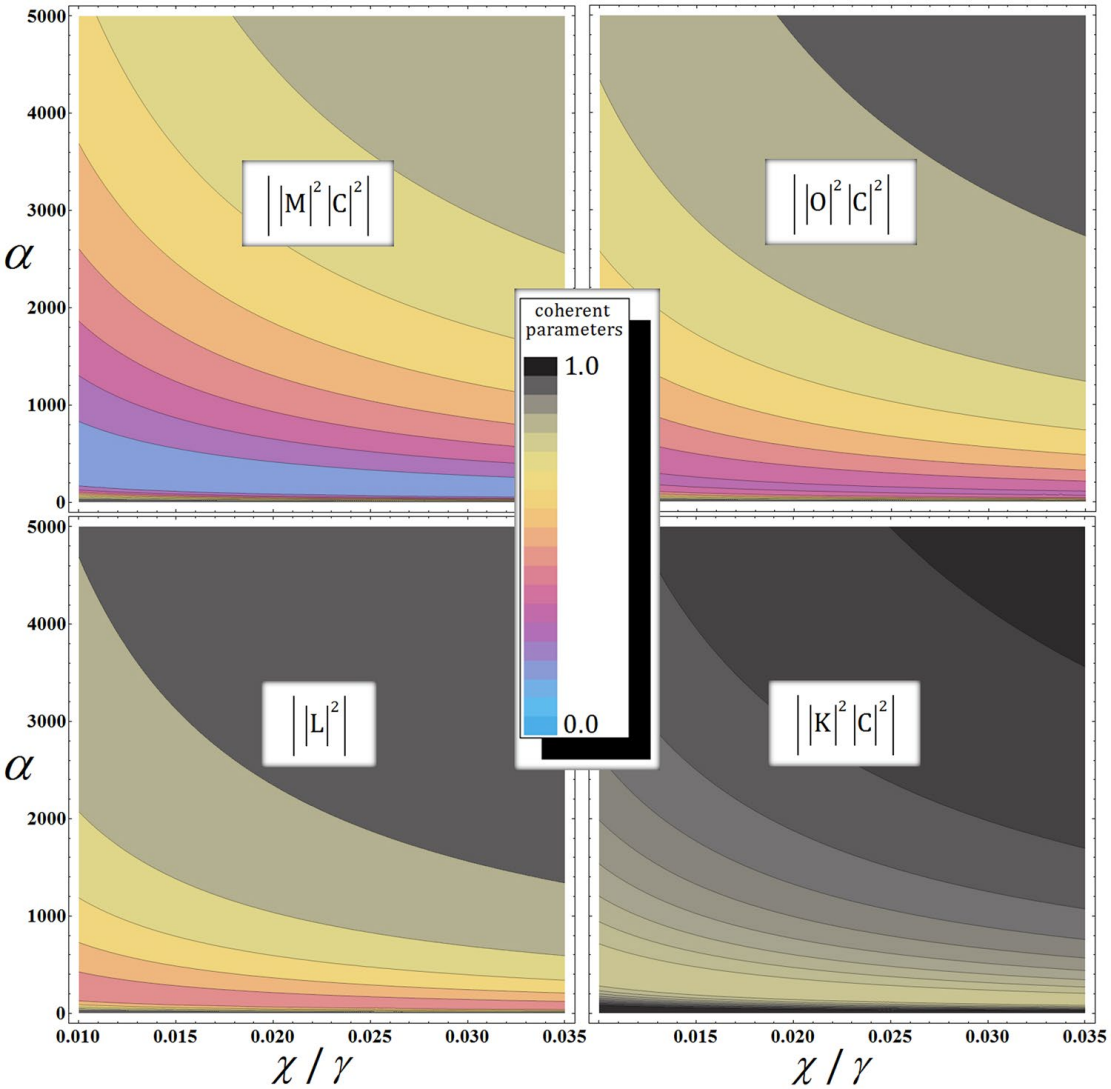
Plots of the coherent parameters in output state *ρ*_PG_ of the PG using XKNL, qubus beams, and PNR measurement for amplitude of the probe beam ($\alpha$) and the $\chi /\gamma$ rate depending on the signal loss of optical fibers, where $N={10}^{3}$, $\alpha \theta =\alpha \chi t\approx 2.5$, and fixed error probabilities ${{\rm{P}}}_{{\rm{err}}}^{{\rm{P}}}={10}^{-3}$.

**Figure 6:**
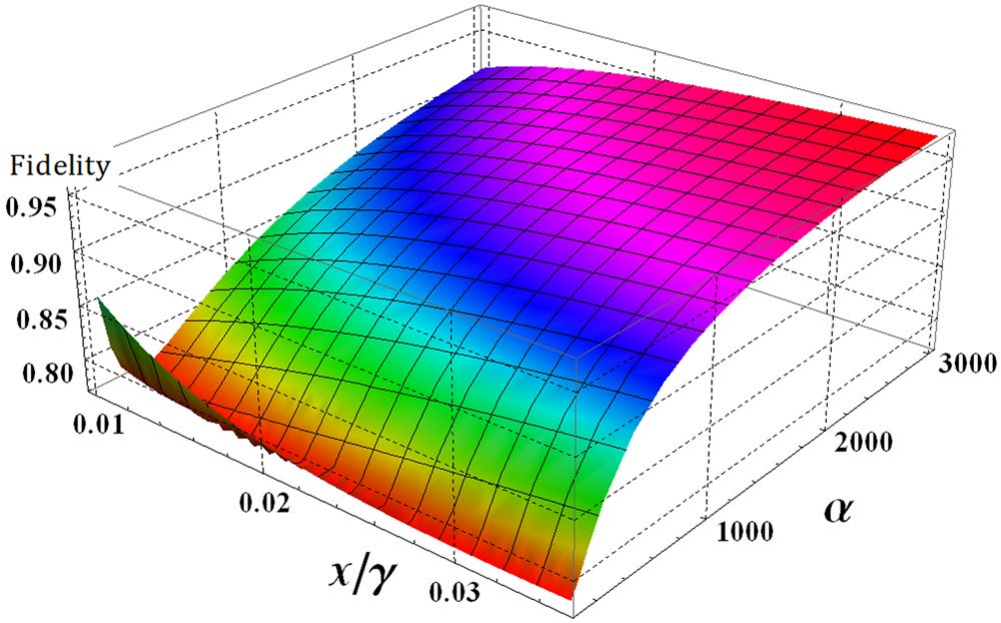
Plot of the fidelity between the ideal, $|{{\boldsymbol{\psi }}}_{{\rm{PG}}}\rangle$, and the practical, *ρ*_PG_, output states of the PG using XKNL, qubus beams, and PNR measurement for amplitude of the probe beam (*α*), and the rate *χ*/*γ* depending on the signal loss of optical fiber, where *N* = 10^3^, *αθ* = *αχt* ≈ 2.5, and fixed error probability ${{\rm{P}}}_{{\rm{err}}}^{{\rm{P}}}={10}^{-3}$. For F → 1 (red color), we should increase the coherent state (probe beams).

Figure 5 shows the absolute values of off-diagonal terms (coherent parameters) in output state ρ_PG_ of the PG for the amplitude of the probe beam (*α*) and the rate for *χ*/*γ*, depending on the signal loss in optical fibers when *α*θ = *αχt* ≈ 2.5 from fixed ${{\rm{P}}}_{{\rm{err}}}^{{\rm{P}}}={10}^{-3}$ in Eq. 4, and N = 10^3^. As described in Fig. 5, if the amplitude of the probe beam gets smaller, then the output state ρ_PG_ of the PG evolves into a mixed state from decreasing the absolute values of coherent parameters. Then, the performance of the PG totally fails from the reduction of the fidelity of the PG, compared with the ideal case output state $|{{\boldsymbol{\psi }}}_{{\rm{PG}}}\rangle$ in Eq. 2. Thus, to maintain output state ${\rho }_{{\rm{PG}}}$ in a pure state (high fidelity), we should increase the amplitude of the coherent state so the absolute values of the coherent parameters (off-diagonal terms in ${\rho }_{{\rm{PG}}}$) approach 1. Moreover, in practical optical fibers at $\chi /\gamma =0.0125$ $(0.364\,\text{dB}/\text{km})$ and $\chi /\gamma =0.0303$ $(0.15\,\text{dB}/\text{km})$ ^64, 65^, we can calculate the fidelity of the PG with $N={10}^{3}$ and $\alpha \theta =\alpha \chi t\approx 2.5$ for ${{\rm{P}}}_{{\rm{err}}}^{{\rm{P}}}={10}^{-3}$, as follows:

15 $${\rm{F}}=|\sqrt{\langle {{\boldsymbol{\psi }}}_{{\rm{PG}}}|{\rho }_{{\rm{PG}}}|{{\boldsymbol{\psi }}}_{{\rm{PG}}}\rangle }|=\frac{1}{2}|\sqrt{1+({|L|}^{2}+{|O|}^{2}{|C|}^{2})+({|K|}^{2}+{|M|}^{2}){|C|}^{2}/2}|,$$

where $|{{\boldsymbol{\psi }}}_{{\rm{PG}}}\rangle$ in Eq. 2 and *ρ*_PG_ in Eq. 13 are the ideal output state and the practical output state, and C, O, L, M, and K are coherent parameters (off-diagonal terms in *ρ*_PG_, Eq. 14). As shown in Fig. 6, output state *ρ*_PG_ of the PG using XKNLs, qubus beams, and PNR measurement is getting closer to the ideal output state $|{{\boldsymbol{\psi }}}_{{\rm{PG}}}\rangle$ (pure state: F → 1) to obtain reliable results of our schemes (A through E) when increasing the amplitude of the probe beam (coherent state) with *N* = 10^3^ and *αθ* = *αχt* ≈ 2.5 for fixed ${{\rm{P}}}_{{\rm{err}}}^{{\rm{P}}}={10}^{-3}$. Table 2 lists the needed *θ* (conditional phase shift: XKNL), the length of the optical fiber, and the fidelities, Eq. 15, between $|{{\boldsymbol{\psi }}}_{{\rm{PG}}}\rangle$ and *ρ*_PG_ with regard to the amplitude of probe beams for *N* = 10^3^ and *αθ* = *αχt* ≈ 2.5 fixed ${{\rm{P}}}_{{\rm{err}}}^{{\rm{P}}}={10}^{-3}$, based on practical optical fiber at $\chi /\gamma =0.0125$ $(0.364\,\text{dB}/\text{km})$ and $\chi /\gamma =0.0303$ $(0.15\,\text{dB}/\text{km})$ ^64, 65^. Table 2 obviously shows that if the output state is mixed state ${\rho }_{{\rm{PG}}}$ (the values of off-diagonal terms are not 1) by photon loss and dephasing, this can approach the pure state as $|{{\boldsymbol{\psi }}}_{{\rm{PG}}}\rangle$ (the off-diagonal terms of 1) by increasing the amplitude of the probe beam, *α* (F → 1 for *α* > 8000). Also, it is difficult to increase the magnitude of the phase shift (XKNL) by EIT^57, 58^ since natural XKNLs are extremely weak: *θ* ≈ 10^−18^ ^60^. However, from the analysis, in Table 2, of the PG under the decoherence effect^41, 42, 52^, when we increase the amplitude of the probe beam to reduce the photon loss rate and the dephasing for high fidelity, the needed magnitude of the induced phase shift, $\theta (=\chi t)$, will be smaller with a fixed error probability, as described in Table 2 (if $\alpha  > 8000$, then $\theta  < 0.00031$ for ${{\rm{P}}}_{{\rm{err}}}^{{\rm{P}}}={10}^{-3}$).

**Table 2:** The fidelities between $|{{\boldsymbol{\psi }}}_{{\rm{PG}}}\rangle$ and *ρ*_PG_ of the PG using XKNLs, qubus beams, and PNR measurement, the magnitude of the phase shift (XKNL), and the required length of the optical fibers due to the amplitude of the probe beam in the optical fiber at 0.364 dB/km corresponding to *χ*/*γ* = 0.0125(=0.0303).

*χ*/*γ*	*α*	*θ*(=*χt*)	Optical fiber length (km)	**Fidelity (F)**
0.0125 (0.364 dB/km)	1000	**0.00250**	**2.387**	**0.8505**
	3000	**0.00083**	**0.792**	**0.9289**
	8000	**0.00031**	**0.296**	**0.9698**
0.0303 (0.15 dB/km)	1000	**0.00250**	**2.387**	**0.9151**
	3000	**0.00083**	**0.792**	**0.9673**
	8000	**0.00031**	**0.296**	**0.9864**

Because we take fixed error probability ${{\rm{P}}}_{{\rm{err}}}^{{\rm{P}}}={10}^{-3}$ *(αθ* =* αχt* = 2.5) and *N* = 10^3^, the condition of the photon decay rate at ${\Lambda }_{t}={e}^{-\gamma t/2}$ will be *γt* = 2.5/0.0125*·α*(=2.5/0.0303*·α*) where *t* = *N*Δ*t* and *θ* =* N*Δ*θ*.

So far, we have analyzed the performance of the multi-photon gate (PG) using a master equation^41, 42, 52^ under the decoherence effect. (The analysis of the HEG using XKNLs, qubus beams, and PNR measurement is equivalent to that of the PG, because the HEG has an almost identical structure as the PG, except for the different length of paths for time-bin encoding). Consequently, we demonstrated that the multi-photon gates (HEG and PG) via XKNL, qubus beams, and PNR measurement are experimentally feasible (weak XKNLs) and robust under the decoherence effect when increasing the amplitude of the probe beam under our analysis. Thus, schemes A through E for distribution and generation of various entanglements can be implemented, having reliable performance when utilizing HEG and PG in practice.

## Conclusion

We proposed several schemes (A through E) to generate and distribute hybrid entanglement and hyperentanglement for two DOFs (polarization and time-bin) using optical multi-qubit gates (HEG and PG), which utilize XKNLs, qubus beams, PNR measurement, and linear optical devices (including time-bin encoders). In two schemes (A and B), verification of the channel and reconstruction of the hybrid entangled state (by users) can be accomplished using an HEG and PCs. And Trent can generate a hyperentangled state with correlations of two DOFs (polarization and time-bin) by attaching a PG and HWPs in scheme (C). For purification under a noisy (bit-flip) channel, only the usage of optical linear devices (including time-bin encoders) in scheme (D), as described by Li *et al*.^31^, can make it possible to purify an entangled state for a single DOF (polarization) from the stored correlation in the time-bin (two photons) in Eq. 8. Furthermore, users in scheme (E) could acquire the expansion of a hybrid entangled state (four photons) from the purified entangled state in scheme (D) via local applications (HEGs and time-bin encoders) and classical communications (the results of PNR measurement in HEGs).

In schemes A through E, HEG and PG using XKNLs, qubus beams, and PNR measurement are experimentally feasible and robust against the decoherence effect when implemented in practice. In particular, through our analysis in Section 4, we demonstrated that the HEG and PG in optical fiber operate reliably and have high efficiency by increasing the amplitude of the probe beam (*α*) in spite of the decoherence effect (photon loss and dephasing). Also, the magnitude of an XKNL (θ) decreases if increasing the amplitude of a probe beam in HEG and PG to obtain robustness against the decoherence effect, as described in Table 2. This means that an HEG and a PG using XKNL can be realized in practical schemes because of the difficulty of the implementation of a large magnitude in XKNLs^59^. Moreover, we can obtain the experimental benefit in our HEG and PG (using only positive conditional phase shifts, θ) because Kok^60^ showed that it is generally not possible to change the sign of the conditional phase shift (−θ). Recently, various propositions about the experimentally feasible technologies in XKNLs have been researched for low error rate and high fidelity. By the adoption of suitable physical systems, the sufficient large strength of XKNL can be provided, such as electromagnetically induced transparency (EIT)^67, 68^, circuit electromechnics^69^, an artificial atom^70^, and three-dimensional circuit quantum electrodynamic architecture^71^. Also, it is possible to enhance the phase shift through detection capability improvement^72^, and phase noise mitigation^73^.

Consequently, we demonstrated from experimental feasibility that schemes A through E can be implemented for the distribution and generation of hybrid entanglement and hyperentanglement via HEG and PG having robustness against the decoherence effect.

## Electronic supplementary material

Supplementary information
