# Supplementary material for: Distribution of hybrid entanglement and hyperentanglement with time-bin for secure quantum channel under noise via weak cross-Kerr nonlinearity
Source: Sci Rep. 2017 Aug 31;7:10208. doi: 10.1038/s41598-017-09510-9 (PMC5579062; doi:10.1038/s41598-017-09510-9)
Supplement: Supplementary file 1 — Supplementary information [file 41598_2017_9510_MOESM1_ESM.doc]

**Title: Distribution of hybrid entanglement and hyperentanglement with time-bin for secure quantum channel under noise via weak cross-Kerr nonlinearity**

Authors: Jino Heo, Min-Sung Kang, Chang-Ho Hong, Hyung-Jin Yang, Seong-Gon Choi, Jong-Phil Hong

**Appendix**

In shown as Fig. 4 (**D: generation four-photon hybrid entanglement**), after two HEGs of Alice and Bob with $|\text{P}^{\text{ }\mp}\rangle$, Eq. 9, and $|R\rangle_{3}|R\rangle_{4}$, the output states $|\text{E}^{\text{ }\mp}\rangle_{1324}$ are given by

(A.1)

Also, we can express $|\text{E}^{\text{ }\mp}\rangle_{1324}$ before PNR measurement, as follows:

(A.2)

Subsequently, after photons (1 and 2) pass through T3s, the final state will be given according to the results of PNR measurements in the HEGs (Trent, Alice, and Bob), as follows:

(A.3)

where we define as $\left\{|0\rangle_{\text{TH}}^{\text{b}}\equiv|-\rangle_{\text{TH}}^{\text{b}},\text{ }|n\rangle_{\text{TH}}^{\text{b}}\equiv|+\rangle_{\text{TH}}^{\text{b}}\right\}$, and $|s^{\text{ 2}}\rangle\equiv|ssl \text{or }sls \text{or }lss\rangle$, $|l^{\text{ }2}\rangle\equiv|lls \text{or }lsl \text{or }sll\rangle$, and $|s^{\text{ 4}}\rangle\equiv|s^{\text{ }2}ss\rangle=|l\times1+s\times4\rangle$, $|s^{\text{ }3}\rangle\equiv|l^{\text{ }2}ss\rangle=|l\times2+s\times3\rangle$, $|l^{\text{ 4}}\rangle\equiv|l^{\text{ }2}ll\rangle=|l\times4+s\times1\rangle$, $|l^{\text{ 3}}\rangle\equiv|s^{\text{ }2}ll\rangle=|l\times3+s\times2\rangle$. And these results (final states) are listed in Table 1.
